# Mechanisms Driving Seasonal Succession and Community Assembly in Sediment Microbial Communities Across the Dali River Basin, the Loess Plateau, China

**DOI:** 10.3390/microorganisms13020319

**Published:** 2025-02-01

**Authors:** Xin Chen, Jing Li, Guoce Xu, Kang Fang, Shun Wan, Bin Wang, Fengyou Gu

**Affiliations:** Key Laboratory of National Forestry Administration on Ecological Hydrology and Disaster Prevention in Arid Regions, Xi’an University of Technology, Xi’an 710048, China

**Keywords:** bacteria, fungi, geographic pattern, seasonal succession, community assembly

## Abstract

Microorganisms are instrumental in river ecosystems and participate in biogeochemical cycles. It is thought that dynamic hydrological processes in rivers influence microbial community assembly, but the seasonal succession and community assembly of river sediments on the Loess Plateau remain unclear. This study used high-throughput sequencing technology (16S and ITS) and the neutral community model to analyze seasonal succession and the assembly processes associated with microbial communities in the Dali River, a tributary of the Yellow River on the Loess Plateau. The results showed that sediment bacterial and fungal community diversity indexes in non-flood season were 1.03–3.15 times greater than those in flood season. There were obvious variations between non-flood and flood seasons in sediment microorganisms. The similarities among all, abundant, and rare microbial communities decreased as geographical distance increased. Proteobacteria (52.5–99.6%) and Ascomycota (22.0–34.2%) were the primary microbial phyla in all, abundant, and rare microbial communities. Sediment ammonia nitrogen, water temperature, and sediment organic carbon significantly affected (*p* < 0.05) the structure of all, abundant, and rare sediment microorganism communities. The ecological networks for the bacterial community of non-flood season and fungal community of flood season had complex topological parameters. The bacterial community in river sediments was driven by deterministic processes, while the fungal community was dominated by stochastic processes. These results expanded understanding about sediment microbial community characteristics in rivers on the Loess Plateau and provided insights into the assembly processes and the factors driving microbial communities in river networks.

## 1. Introduction

Watershed ecosystems play crucial roles in preserving biodiversity, providing aquatic resources, and advancing economic growth [1]. Nevertheless, these ecosystems are easily affected by an extensive variety of human activities, such as excessive agricultural fertilization and the discharge of untreated wastewater, and global climate change, which has led to extreme temperature fluctuations and an increase in disasters such as floods [2,3]. These factors can effectively reduce soil quality, increase silt accumulation in riverbeds, and contaminate water resources. Due to increasing pressures from the expansion of human societies and associated development goals, assessing the health and resilience of watershed ecosystems by investigating water quality indices and land use patterns can be challenging [4]. Microorganisms are the most numerous, varied, and complex life forms on the Earth, playing crucial roles in ecosystem services within water environments [5]. They engage in fundamental processes, such as nutrient cycling and the decomposition of waste, which support and preserve the health and equilibrium of river ecosystems [6,7]. However, spatial variability among the physicochemical properties within a river influence microorganism diversity and community structure. The diversity of microorganisms serves as a biological indicator of an ecosystem’s functionality and can be employed to assess the ecological vitality of watershed ecosystems [2]. Hence, it is essential to comprehend the distribution patterns of microbes within these ecosystems and to comprehend the mechanisms that drive microbial diversity.

Studies on environmental change and its effects on the underlying factors that shape microbial community structure and function and their corresponding ecological mechanisms have become increasingly important in microbial ecology [8,9]. Microbial communities of river sediments have been impacted by sediment accumulation, deposition, and erosion throughout sedimentary history [10]. Previous studies surveyed the comparative effects of environmental variables on riverine microbial assemblages. Notably, in the Yellow River of China, research has revealed that the bacterial community in water is affected by pH, dissolved organic carbon (DOC), and suspended particles, while the bacterial community in sediments is mainly affected by pH, nitrate nitrogen, and water content [11]. Research has demonstrated a significant correlation between bacterial communities and various factors, including annual precipitation within the water column, sediment pH levels, and nitrogen sources, in the aquatic ecosystems fed by glacier [12]. Microbial community composition fluctuates across rivers in response to diverse environmental pressures, including seasonal shifts and natural disturbances. Recent innovations in high-throughput technologies and cost reductions have meant that microbiome analyses can now be used to monitor microbial standard and diversity in aquatic environments [12].

A microbial “rare biosphere” was confirmed by high-throughput sequencing and increased sampling, consisting of numerous species represented by a few individuals [13]. These elusive microorganisms perform unique metabolic activities and possess crucial specialized metabolic functions associated with the cycling of certain elements [14]. This suggests that rare microbes can work as a repository of reproductive potential. Abundant microbes that have been explored, in contrast, to a greater degree, seem to hold a critical position in the ecosystem [15]. Considering the importance of rare and rich taxa, studying their biological function and ecological geographical pattern in future research is important to provide valuable insights [13]. Nevertheless, it is not known whether rare microbiomes have similar biogeographic features to abundant microbiomes.

Identifying the mechanisms driving microbial community assembly is crucial when attempting to understand microbial diversity, distribution, function, biogeographical patterns, and successional processes [16,17]. Microbial community assembly is known to be driven by both deterministic and stochastic processes [18,19]. Recent studies on microbial community assembly mechanism were studied, but these studies have focused on lakes and wetlands such as low liquidity ecosystems [20,21,22], whereas river environments and their microbial communities are subject to more complex and dynamic changes than low-mobility ecosystems [23]. At present, the seasonal succession of microbes in Yellow River deposition and accumulation mechanism research is limited. Research on seasonal succession and the assembly processes associated with sediments microbes of the middle of the Yellow River will improve understanding about the mechanisms controlling microbial diversity and ecological functions in these riverine ecosystems.

In this study, 16S rRNA and ITS sequencing techniques were applied to investigate the bacterial and fungal communities in sediments from different periods (non-flood and flood seasons) in the Dali River, respectively. Particularly, the following hypotheses were proposed for the study of microbial community diversity, geographical pattern, and community mechanism: (1) microbial communities showed seasonal dynamics; (2) all, abundant, and rare microorganisms had similar geographical distribution patterns in two seasons; (3) the influence of environmental factors on microbial communities were different; and (4) bacterial and fungal community assembly mechanisms were dissimilar.

## 2. Materials and Methods

### 2.1. Study Area

**Figure 1:**
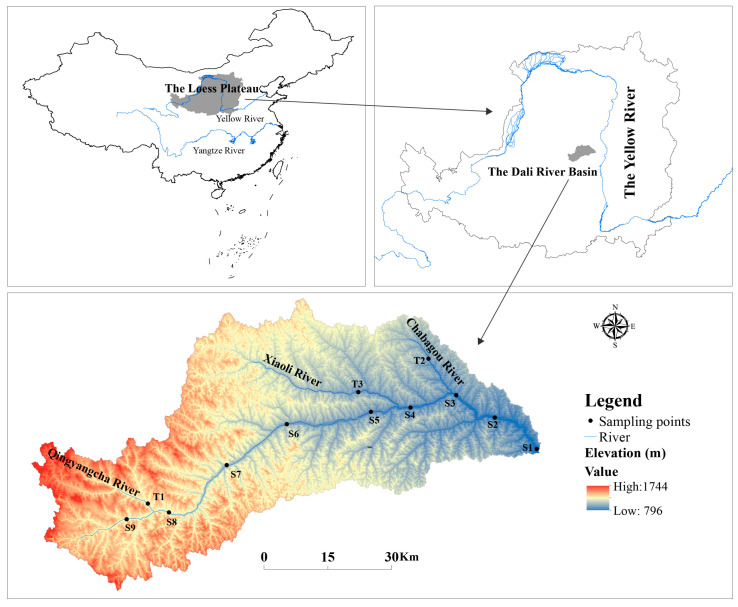
Study area.

The Dali River (DLR) is the largest tributary of the Wuding River and a secondary tributary of the Yellow River, China (109°14′ E~110°13′ E, 37°30′~37°56′ N) (Figure 1). It originates in Baiyu Mountain, Jingbian County, Shaanxi Province. The DLR flows east through Suide County to the Wuding River and its tributaries include the Chabagou, Xiaoli, and Qingyangcha Rivers. The length of the main stream is 170 km, with a drainage area covering 3906 km^2^. The basin is located in the temperate continental monsoon climate zone, the annual average precipitation is 380 mm, and precipitation mainly occurs between July and September.

### 2.2. Sampling Process and Measurement of Physicochemical Properties

There were 12 sample sites in the DLR basin. Nine were situated in the main stream area and three were positioned in the tributary area (Figure 1 and Table S1). Samples were collected twice, one in October 2020, during non-flood season (dry season), and the other in August 2021, during flood season (wet season). The collection and determination methods of water and sediment samples were mentioned in the study of Chen et al. [24], as detailed in S1 (Supplementary Materials).

### 2.3. DNA Extraction and PCR Amplification

DNA was extracted from the sediment samples using the E.Z.N.A.^®^ soil DNA kit. The nanodrop method was applied for determining the amount of extracted DNA and the 2% agarose gel electrophoresis method to evaluate the quality of the extracted DNA. Primer sequences 341F (5′-CCTACGGGNGGCWGCAG-3′) and 805R (5′-GACTACHVGGGTATCTAATCC-3′) were utilized for PCR amplification of the V3-V4 hypervariable region fragments of the 16S rRNA gene from the bacteria in the sediment. Additionally, primer sequences ITS1FI2 (5′-GTGARTCATCGAATCTTTG-3′) and ITS2 (5′-TCCTCCGCTTATTGATATGC-3′) were employed to amplify the V1-V2 hypervariable region fragments of the ITS gene from the fungi. The PCR amplification and sequencing processes were carried out by Xi’an Baikeli Genetic Technology Co., Ltd., Xi’an, China.

This research work identified all, abundant, and rare sediment microbial communities using previously established methodologies [25]. The OTUs’ relative abundance is more than 0.1% for the rich microbial community, and the relative abundance of the OTUs was less than 0.01% for the rare microbial communities.

### 2.4. Statistical Analyses

QIIME2 v.2022.8 software was used to remove the filtered error sequence [26]. The sequence was classified as an operational unit (OTU) using a 97% recognition threshold. The QIIME2 software was utilized to compute alpha diversity indexes, diversity was measured by the Shannon index, and richness was measured by the Chao1 index. SPSS v.24.0 was used for date analysis, and the Wilcoxon rank sum test of independent samples was employed for evaluating the characteristic differences in the physical and chemical properties of the water and sediments, as well as the changes in microbial community α diversity at different periods. Using Bray–Curtis distances, principal coordinates analysis (PCoA) was employed to investigate alterations in beta diversity among sediment microbial communities in two periods. To ascertain the statistical significance of differences between different seasons, a non-parametric multivariate analysis of variance known as PerMANOVA (Adonis) was conducted [23]. Significant biomarker taxa were identified through Linear Discriminant Analysis (LDA) [26]. Additionally, the Mantel test was utilized to study the relationship between microbial community composition and the environmental factors, and the relationship between community function and the environmental factors. The Bray–Curtis similarity matrix of the sediment microbial community was calculated using the “vegdist” function of the vegan package, and the “distm” function of the geosphere package was used to calculate the geographical distance of each sampling point. The correlation between community similarity and geographic distance was calculated using the “lm” function (ordinary least-square linear regression) [27].

Redundancy analysis (RDA) was employed to quantify how physicochemical indicators of water and sediment, as well as interspecies interactions, influenced microbial communities. The selection of RDA and CCA was determined by DCA, and the first axis of the lengths of gradient was greater than 4.0. If it was less than 3.0, RDA was used. Before RDA or CCA, environmental factors with variance inflation factors (VIFs) less than 10 were selected.

To eliminate false positives and obtain more reliable results, genus-level species were selected for network analysis. Spearman correlation was performed on the filtered data sets, and only the highly correlated and meaningful correlations (|R| ≥ 0.8, *p* < 0.05) were saved in the network and then imported into Geohi0.9.7 for the calculation and visualization of network topology parameters. We predicted the function of bacterial communities through the FAPROTAX database [3] and the functional composition of fungal communities using PICRUSt2 [28]. The differences in community function were analyzed by STAMP v.2.1.3 software.

Sloan neutral community models (NCMs) were applied to quantify the potential contribution of stochastic processes during microbial community assembly [25,29]. The NCM used nonlinear least squares to obtain the best fit between frequency and relative abundance of OUTs, R^2^ represented the fit degree of the model, and m represented the mobility of the community. C-scores were calculated based on the “EcoSimR” package and the effect of the standard size (SES) to assess deterministic process in the assembly of the microbial community and the relative importance of the stochastic process [29]. The observed index of C-score and the simulated index were compared to see if there was any significant difference between the two values. If no significant difference was observed, it indicated that stochastic processes were primary. If there was a significant difference, it suggested that deterministic processes were dominant. The C-score was evaluated shaped by 1000 cycles. In addition, standardized effect size (SES) obtained by C-score analysis could further determine the pattern of species coexistence in the community: an SES value > 2 indicated that the community was competitive. If the SES value was <−2, it indicated that the community behaved as a facilitation. If the SES value was −2~2, it suggested that the community showed a stochastic coexistence pattern, that is, the community as a whole showed a neutral role [30]. The “spaa” package in R was operated for calculating Levin’s niche breadth index to illustrate a deterministic and stochastic process model and its contribution to the particular habitat microbial communities [25]. The drawing was completed by Arcgis10.2, Origin2021, Gephi0.7, and R 4.3.0.

## 3. Results

### 3.1. Diversity and Distance-Decay Patterns of the Sediment Microbial Communities

**Figure 2:**
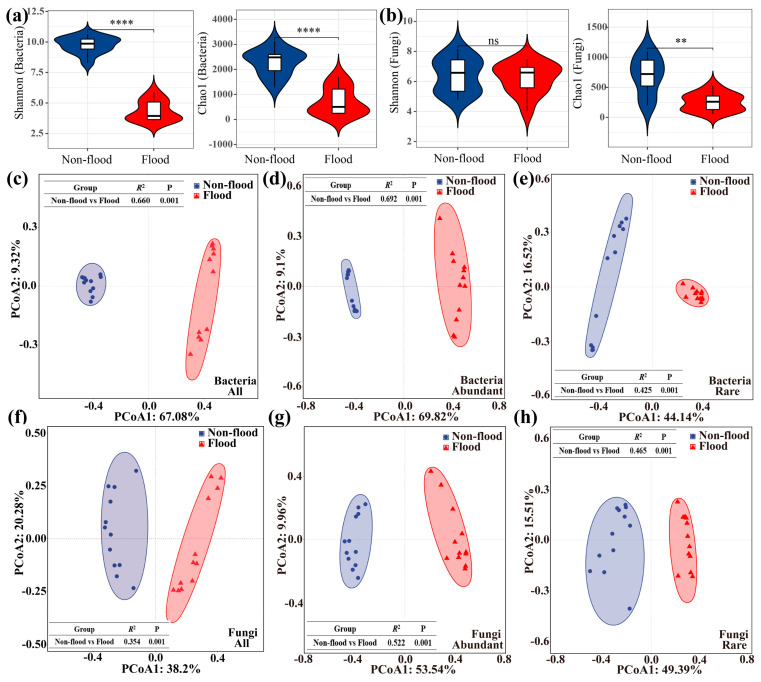
Diversity and distribution patterns of microbial community during non-flood and flood seasons. (**a**) Diversity of bacterial community. (**b**) Diversity of fungal community. (**c**–**h**) PCoA and PerMANOVA showing the variation in the microbial community (OTU levels) based on “Bray–Curtis” distance. Notes: ** indicated 0.01 level of significance; **** indicated 0.0001 level of significance; ns indicated a significance level greater than 0.05.

Alpha diversity, which measures microbial diversity within each group, was estimated using the Shannon and Chao1 indexes. The findings showed that sediment microbial community diversity and richness were greater during non-flood season than flood season (Figure 2a,b). The fungal diversities during non-flood and flood seasons appear not to be as different as the bacterial diversities.

PCoA and PerMANOVA based on the microbial communities were used to classify the samples, and the species diversity of differences between the groups was determined (Figure 2c–h). The PCoA results showed that the closer the distance between each group of samples, the higher the similarity. The two main axes explained 76.40% of the difference among the all-bacteria communities (Figure 2c) and the sediment bacterial samples in different seasons were distributed on both sides, indicating that sediment all-bacteria community structures in different seasons were significantly different. The two axes explained 58.48% of the difference among the all-fungi communities (Figure 2f), indicating that the all-fungi community structures in different seasons were also significantly different. The larger the R^2^ produced by PerMANOVA, the greater the explanation of alterations between the groups, and the more significant the variation between the groups. The PerMANOVA results showed significant differences between seasons in all-bacteria communities (R^2^ = 0.66, *p* = 0.001). Similarly, all-fungi communities varied significantly across seasons (R^2^ = 0.354, *p* = 0.001). These results were consistent with the PCoA. Similarly, the results for the abundant microbial communities (Figure 2d,e) and rare microbial communities (Figure 2g,h) also showed that there was a large difference between non-flood and flood seasons.

**Figure 3:**
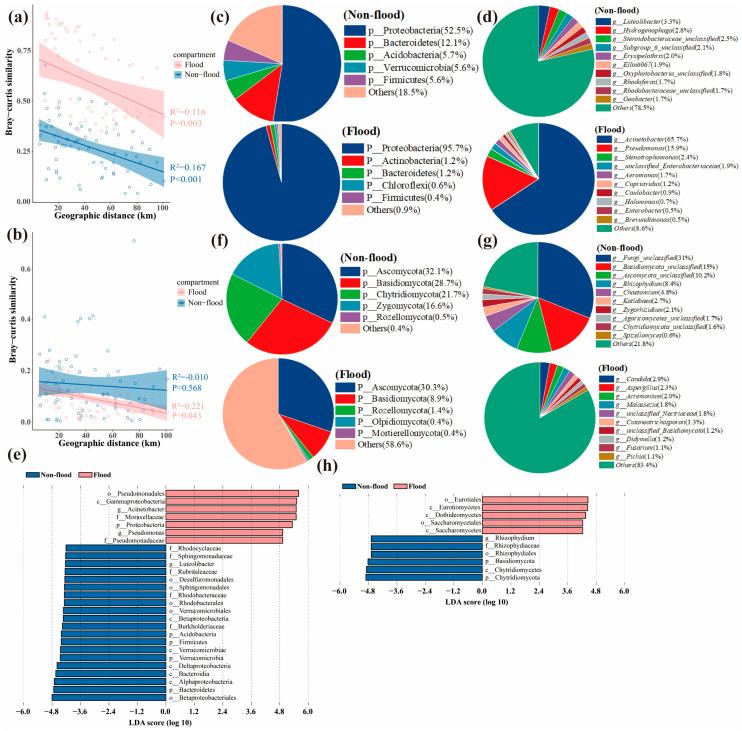
Community similarity, composition, and biomarker of microbial community during non-flood and flood seasons. (**a**,**b**) Linear regression between geographic distance and community similarity (1-“Bray–Curtis” distance) during non-flood and flood seasons. Solid lines indicate the ordinary least-square linear regression. (**a**) Bacteria. (**b**) Fungi. (**c**) Taxonomic composition of bacterial community at phylum level. (**d**) Taxonomic composition of bacterial community at the genus level. (**e**) Variation in taxonomic composition of bacterial community. (**f**) Taxonomic composition of fungal community at phylum level. (**g**) Taxonomic composition of fungal community at genus level. (**h**) Variation in taxonomic composition of fungal community.

**Figure 4:**
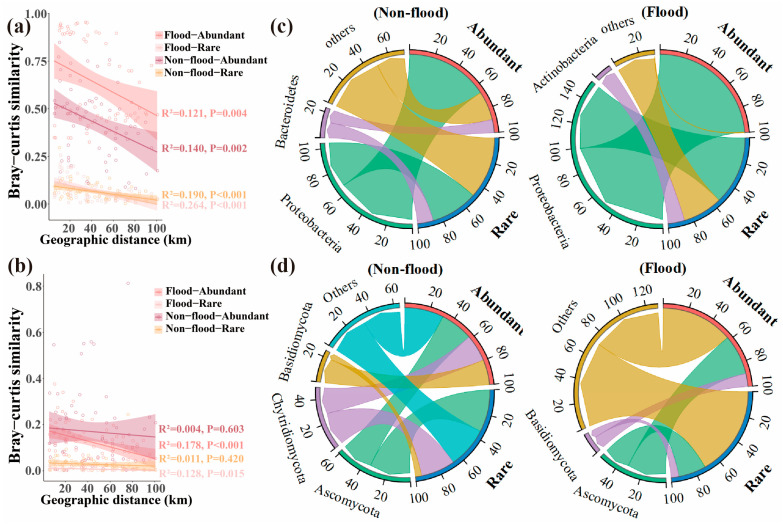
Community similarity and composition of abundant–rare microbial community during non-flood and flood seasons. (**a**,**b**) Linear regression between geographic distance and abundant–rare community similarity (1-“Bray–Curtis” distance) during non-flood and flood seasons. (**a**) Abundant–rare bacteria. (**b**) Abundant–rare fungi. (**c**) Taxonomic composition of abundant–rare bacterial community at phylum level. (**d**) Taxonomic composition of abundant–rare fungal community at phylum level.

The distribution patterns of the microbial communities were evaluated using the distance-decay pattern. The all-bacteria and all-fungi community structures showed spatial changes that followed a distance-decay pattern in different seasons (Figure 3a,b). Notably, there was no significant distance-decay pattern for sediment fungal communities (all, abundant, and rare) during non-flood season (Figure 3b and Figure 4b). A similar distance-decay pattern was registered for abundant and rare sediment microbial communities, suggesting that community structures also showed spatial variation (Figure 4a,b).

### 3.2. Structure of the Sediment Microbial Community

The classification and composition of microbial phyla in the sediments from different hydrological periods were used to identify the top five species based on relative abundance. The remaining species were merged into “Others”. In the all-bacteria communities, the most abundant phyla in non-flood season were Proteobacteria (52.5%), Bacteroidetes (12.1%), and Acidobacteria (5.7%), whereas the most abundant phyla in flood season were Proteobacteria (95.7%), Actinobacteria (1.2%), and Bacteroidetes (1.2%) (Figure 3c). The top ten dominant bacterial genera in the bacterial communities are shown in Figure 3b. The LDA and Kruskal–Wallis tests showed that there were 20 and 7 biomarkers in different seasons (Figure 3e). Betaproteobacteriales (order level) was the most significant biomarker group in non-flood season, while Pseudomonadales (order level) was the most significant biomarker group in flood season (*p* < 0.05) (Figure 3e). Comparable to the all-bacteria community, Proteobacteria also had the largest OUT abundances in both rare and abundant bacterial communities at 37.75% to 99.58% in different seasons (Figure 4c).

The top three phyla in the all-fungi communities during non-flood season were Ascomycota (32.1%), Basidiomycota (28.7%), and Chytridiomycota (21.7%), whereas the top three phyla in flood season were Ascomycota (30.3%), Basidiomycota (8.9%), and Rozellomycota (1.4%) (Figure 3f). The top ten dominant fungal genera in all-fungi communities are shown in Figure 3g. The LDA and the Kruskal–Wallis tests results showed that there were 6 and 5 biomarkers in different seasons (Figure 3h). Chytridiomycota (phylum level) was the most significant biomarker group in non-flood season, and Eurotiales (order level) was the most significant biomarker group in flood season (*p* < 0.05) (Figure 3h). Similarly to the all-fungi communities, Ascomycota also had the largest OUT abundances in both the abundant and rare fungal communities at 26.69% to 34.18% in different season (Figure 4d).

### 3.3. Environmental Factors Affecting Microbial Communities

The characteristic analysis of the DLR showed that DO, T, W-AN, W-TP, S-TP, S-AN, and S-TOC had significant differences in two seasons (*p* < 0.05) (Figure S1). Among them, the DO, W-AN, S-AN, and S-TOC in non-flood season were 1.27, 2.14, 3.06, and 7.50 times that of those during flood season, respectively. The T, W-TP, and S-TP in flood season were 1.74, 5.6, and 1.04 times that of those during non-flood season, respectively.

**Figure 5:**
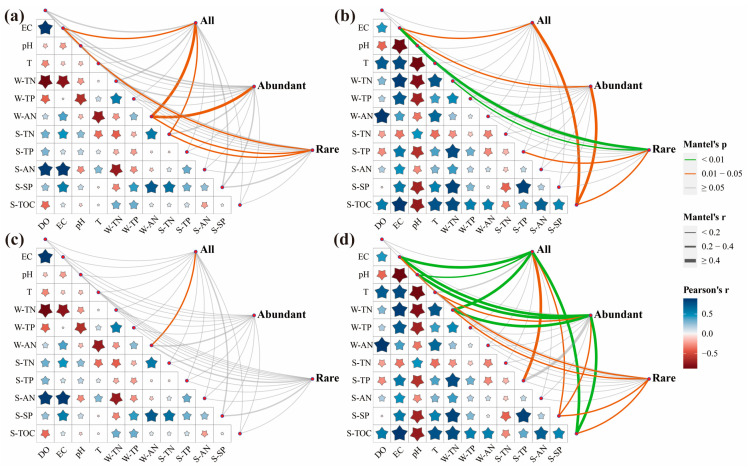
Environmental drivers of the microbial community by Mantel test (OUT level). (**a**) Bacteria (non-flood). (**b**) Bacteria (flood). (**c**) Fungi (non-flood). (**d**) Fungi (flood). Notes: the larger the shape of start, the greater the correlation coefficient.

**Figure 6:**
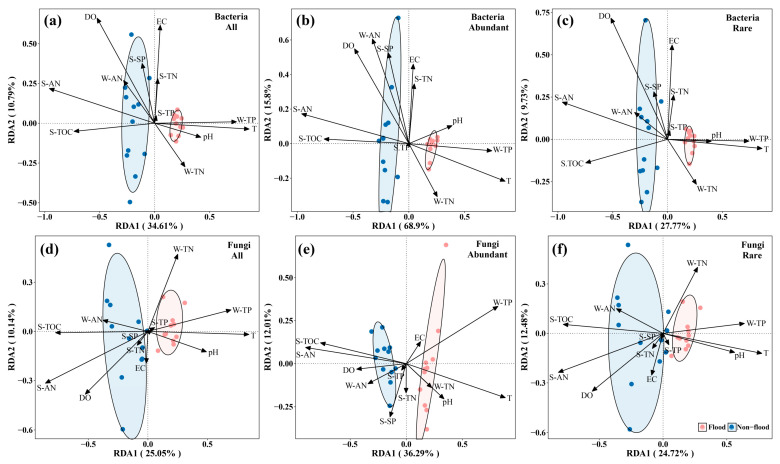
Redundancy analysis (RDA) of microbial communities and environmental factors during non-flood and flood seasons (genus level).

They were shaped by the OTUs and environmental factors on the Mantel test to check for two seasons of microbial community and the relationship between the environmental factors (Figure 5). The correlation between the microbial community and environmental factors was higher in flood season than non-flood season, and the correlation between the bacterial community and environmental factors was higher than that between the fungal community. RDA based on the genus-level species and environmental factors was also conducted to distinguish environmental factors that significantly impacted microbial communities (Figure 6). For all-bacteria communities, S-AN (R^2^ = 0.923), T (R^2^ = 0.708), DO (R^2^ = 0.680), W-TP (R^2^ = 0.530), S-TOC (R^2^ = 0.517), and EC (R^2^ = 0.365) were important environmental factors driving structural changes (*p* < 0.05) (Figure 6a), whereas S-AN (R^2^ = 0.932), T (R^2^ = 0.776), S-TOC (R^2^ = 0.562), W-TP (R^2^ = 0.540), DO (R^2^ = 0.512), W-AN (R^2^ = 0.440), and EC (R^2^ = 0.282) were important environmental factors driving structural change in the abundant bacterial community (*p* < 0.05) (Figure 6b) and S-AN (R^2^ = 0.937), DO (R^2^ = 0.753), T (R^2^ = 0.720), S-TOC (R^2^ = 0.559), and W-TP (R^2^ = 0.533) were important environmental factors driving structural change in the rare bacterial community (*p* < 0.05) (Figure 6c).

In the all-fungi communities, S-AN (R^2^ = 0.757), T (R^2^ = 0.654), S-TOC (R^2^ = 0.546), and W-TP (R^2^ = 0.456) were important environmental factors driving structural change (*p* < 0.05) (Figure 6d), whereas S-AN (R^2^ = 0.765), T (R^2^ = 0.756), W-TP (R^2^ = 0.748), and S-TOC (R^2^ = 0.560) were important environmental factors driving structural change in the abundant fungal community (*p* < 0.05) (Figure 6e) and S-AN (R^2^ = 0.631), T (R^2^ = 0.565), and S-TOC (R^2^ = 0.558) were important environmental factors driving structural change in the rare fungal community (*p* < 0.05) (Figure 6f).

These results suggested that S-AN, T, and S-TOC were the significant, common factors influencing variation in all, abundant, and rare sediment microbial community structures. Furthermore, in abundant microbial communities, physicochemical properties explained the variation better than in total and rare microbial communities.

### 3.4. Symbiotic Networks and Functional Prediction of Microbial Communities

**Figure 7:**
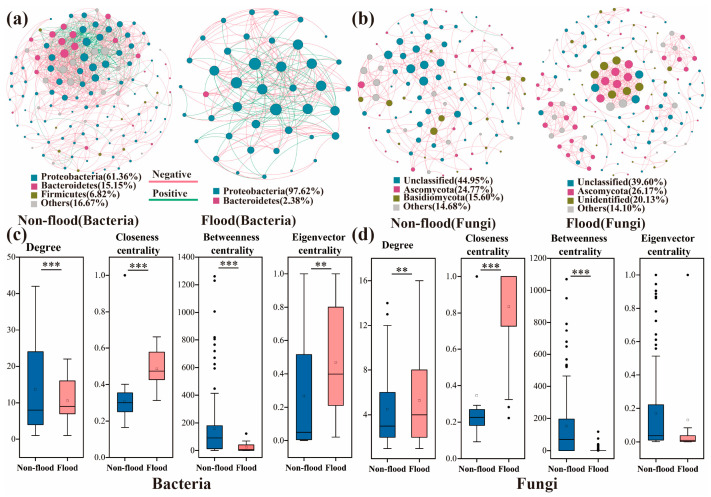
Co-occurrence network analysis. (**a**,**b**) Co-occurrence networks during non-flood and flood seasons. (**c**,**d**) Node-level topological characteristic parameters. Notes: ** indicated 0.01 level of significance; *** indicated 0.001 level of significance.

The co-occurring network at the genus level in different seasons indicated that bacterial groups involved in the network were mainly Proteobacteria (61.36–97.62%) (Figure 7a) and fungal groups involved in the network were mainly Ascomycota (24.77–26.17%) (Figure 7b). The co-occurring bacterial and fungal networks in different seasons were significantly different and had different network topologies (Table 1; Figure 7c,d). The ecological network of the bacterial community had more nodes and edges in non-flood season. In contrast, fungi results showed that there were more nodes and edges during flood season than non-flood season. This indicated that more complex interconnections and network structures existed between the bacterial communities of the non-flood season and fungal communities of the flood season. The modularity of the bacterial co-occurrence network model was 0.377 in non-flood season and 0.330 in flood season (<0.4), and modularity for the fungal co-occurrence network model was 0.678 in non-flood season and 0.833 in flood season (Table 1), which suggested that fungi had a more modular structure. The degree and betweenness centrality values for the bacteria were significantly higher during non-flood season, whereas closeness and eigenvector centrality values were significantly greater during flood season (Figure 7c). In contrast, the degree and closeness centrality values for fungi were significantly greater during flood season, whereas betweenness centrality was significantly higher during non-flood (Figure 7d).

A total of 71 functions were annotated for sediment bacteria, which suggested that the DLR has a high functional diversity compared to other rivers [31]. Among them, 70 functions were annotated during non-flood season and 61 during flood season (Figure S2). Chemoheterotrophy and aerobic_chemoheterotrophy were the main functions annotated to the bacteria (Figure S4). The primary functional layers of the fungal community covered four classes (Figure S3). In order of abundance, they were biosynthesis (52.65–65.14%), generation of precursor metabolites and energy (20.54–27.92%), degradation/utilization/assimilation (14.41–117.83%), and glycan pathways (0.37–1.60%) (Table S2). Among them, the biosynthesis was significantly greater during non-flood season, while the other three functions were significantly lower during non-flood season (Table S2). In addition, Mantel test analysis indicated that there was no significant correlation between the microbial community function and environmental factors (Figure S5).

### 3.5. Sediment Microbial Community Assembly and Ecological Niche

**Figure 8:**
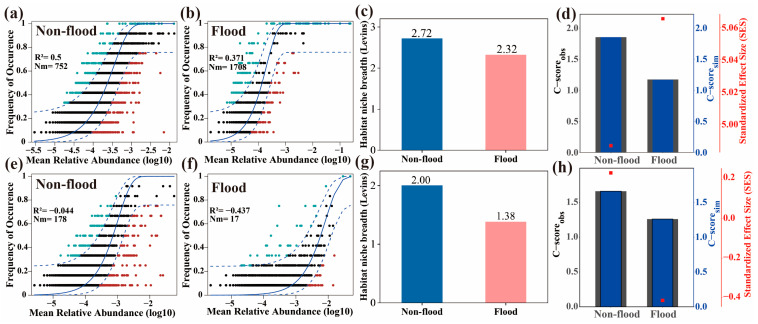
Ecological processes shaping the sediment microbial community assembly at Dali River. (**a**,**b**,**e**,**f**) Predicted frequency of bacterial (**a**,**b**) and fungal (**e**,**f**) communities during non-flood and flood seasons in Dali River. The solid blue line is the best fit to the neutral community model (NCM), and the dashed blue line indicates 95% confidence intervals around the NCM prediction. OTUs that occur more or less frequently than predicted by the NCM are shown in green and red, respectively. (**c**) Bacteria and (**g**) fungi: comparison of mean habitat niche breadth (Levins) for all taxa between non-flood season and flood season. (**d**,**h**) C-score metric using null models.

The neutral community models’ degrees of fitting for the sediment all-bacteria communities in different seasons were low (R^2^ ≤ 0.5), revealing that stochastic process was less involved in bacterial community assembly (Figure 8a,b). The R^2^ value for the NCM was greater in non-flood season, which presented that the impact of the stochastic process on the bacterial community was greater in non-flood season. In addition, the Nm value for microbial groups during flood season (Nm = 1708) was higher than in non-flood season (Nm = 752), which suggested that the species dispersal limitation for sediment bacteria was higher during non-flood season [23]. The C-score analysis results for bacterial communities during different seasons (Figure 8d, Table S3) indicated that observed values were significantly higher than simulated values produced by the NCM and that assembly modes for the bacterial communities were non-random. The SES values were all greater than two, which further indicated that deterministic process dominated sediment bacterial community assembly in the DLR.

In contrast, there was no fitting relationship between the fungal community and the NCM (R^2^ < 0) (Figure 8e,f), suggesting that fungal community assembly was not driven by randomness factors [32]. The C-score analysis results (Figure 8h, Table S3) showed that the observed values for the fungal community in both seasons were not significantly higher than the corresponding simulated mean values produced by the NCM (*p* > 0.05), which suggested that fungal community assembly mode was random. Furthermore, SES values for the two seasons were both greater than –2 and less than 2, which further suggested that stochastic processes dominated fungal community assembly in the DLR. Additionally, higher habitat niche breadths were observed for non-flood season than flood season (Figure 8c,g).

## 4. Discussion

### 4.1. Seasonal Dynamics Affect Sediment Microbial Community Diversity

In recent years, a great deal of research has been carried out on microbial community diversity from the source area, main stream, estuary, and reservoir areas of the Yellow River in sediments [33,34,35,36], but there have been few reports on the sediment microbial communities in tributaries. The diversity (α and β) and taxonomic composition of microbial communities in this study showed seasonal variations (Figure 2 and Figure 3e,h). In addition, PCoA results showed temporal variations in microbial community structure, revealing that microbial communities can come together in the same season (Figure 2c–h). Similarly, river microbial communities have been reported to remain relatively stable from season to season, with a seasonal cycle [37]. In addition, planktonic bacteria communities in estuarine ecosystem and microeukaryotic communities in river communities have similar observations [23,38]. These seasonal differences might be due to effects seasonal changes, such as velocity, temperature, and other environmental factors, have the interactions between microbial species. These effects may trigger more intense competition between species and cause microbial communities to compete for resources. This may explain why there are significant community structure differences in river sediments across different seasons.

The α diversity indexes for the microbial communities were greater in non-flood season (Figure 2a,b), which was similar to Liu et al. [39] and Zhou et al. [38]. However, the results differed from those of microbial community diversity in Tingjiang River, China [23]. The main reasons for differences between the seasons could be as follows: first, the river in non-flood season was relatively low, and the flow rate for the growth of microorganisms provided a stable environment, increased the microbial proliferation, and reduced the competition. This resulted in the improvement of microbial diversity in the non-flood season. Second, more industrial and agricultural sewage and nutrients from the DLR basin entered the river in surface runoff during the flood season, which enriched some bacteria, but reduced the diversity of the sediment microbial community in flood season. Third, the sediment had a higher organic carbon content in non-flood season and this have led to increased bacterial diversity in the sediment [40].

Distance-decay patterns were significant in different seasons, but the relationship was weaker during flood season compared to non-flood season (Figure 3a and Figure 4a). The long-distance relationships between the microeukaryotic community in subtropical rivers and the phytoplankton community in Taihu Lake Basin have also been shown to be weak in flood season [23,25]. Moreover, the symbiotic networks results indicated that interactions in the bacterial community network were weaker during flood season than non-flood season (Figure 7a and Table 1), which was in keeping with the findings reported for estuarine ecosystems and the Taihu basin [25,38]. However, significant distance-decay patterns were only registered in flood season and distance-decay patterns were stronger during flood season (Figure 3b and Figure 4b). The symbiotic networks result also showed that fungal interactions were stronger in flood season (Figure 7b and Table 1). This suggested that the increased precipitation in flood season could trigger a decrease in microbial community diversity by establishing close fungal network interactions rather than close bacterial network interactions.

### 4.2. Seasonal Dynamics of the Dominant Phyla in the Microbial Community

The dominant bacterial groups in the DLR sediments were Proteobacteria, Bacteroidetes, Actinobacteria, and Acidobacteria (Figure 3c). These dominant groups contained a lot of genes related to carbon, nitrogen, phosphorus, and sulfur cycles. All of these processes are involved in the production of extracellular polymers through biofilm formation and can promote substance transformation [41,42]. Proteobacteria were most abundant among the sediment bacteria and members of the Proteobacteria were involved in degradation and metabolism processes [33,43]. Affiliates of the Bacteroidetes played a vital part in the deposition of sediment, such as organic matter decomposition and fermentation, which were an important step in the anaerobic food web [44]. Protobacteria and Bacteroidetes were common freshwater phyla and were dominant groups in sediment bacterial communities [45,46]. Actinobacteria were vastly dispersed in aquatic ecosystems. They played vital roles in humus formation because they contributed to decomposition and nutrient cycling of complex substances in dead fish and algae [47]. It had also been reported that Acidobacteria were abundant in bacteria and had a substantial impact on the biogeochemical and nutrient cycles associated with organic matter decomposition in sediments [48]. Furthermore, these dominant phyla show strong environmental adaptability and material utilization abilities, which allow them to occupy dominant positions in the complex river environment.

The primary fungal phyla in DLR sediments were Ascomycota, Basidiomycota, Chytridiomycota, and Rozellomycota (Figure 3f). In the sediment fungal communities of the Songhua and Yangtze Rivers (China), Ascomycota and Basidiomycota were also the primary phyla [49,50]. They were important decomposers in ecosystems because they degraded organic matter and produced beneficial metabolites [51]. Basidiomycetes were mostly saprophytes, and their reactions to environmental stress was relatively stable [52]. Basidiomycetes were particularly known for their resistance to environmental stress and may be essential to maintaining the stableness of aquatic ecosystems [53]. Chytridiomycota were important in aquatic environments because they primarily fed on dead aquatic plants and were prey for zooplankton [54]. Members of Rozellomycota were regarded as the most basal clade of fungi and were found in many kinds of ecosystems [53,55]. Fungi served important roles as decomposers, parasites, and go-betweens for organisms that feed on detritus in various environments [56]. Nevertheless, understanding of their distribution and ecological functions was still restricted, particularly in aquatic settings [57].

### 4.3. Influencing Factors of Microbial Community

The environmental properties examined in this study identified S-AN, T, DO, W-TP, and S-TOC as notable factors influencing the bacterial variations observed across all, abundant, and rare sediment community structures. S-AN, T, and S-TOC significantly influenced all, abundant, and rare fungal community structure variations (Figure 6). In this study, S-AN, T, DO, W-TP, and S-TOC were significantly diverse from the samples (Figure S1). Carbon, nitrogen, and phosphorus were essential elements for microbial growth, so it was not surprising that their levels significantly influenced the microbial communities. Water pH significantly affected microbial communities and that pH can alter the microbial community structure by converting environmental factors and functional microorganisms [25,58]. However, pH had little impact on the microbial community in the DLR.

Water temperature was one of the important elements altering the water environment and had different degrees of influence on biological activity and the ion concentration in aquatic environment. The DO level can indirectly reflect the self-purification ability of water and directly affect aerobic microorganisms. The DO in non-flood season (11.11 ± 2.52 mg/L) was significantly greater than flood season (8.73 ± 1.50 mg/L), which suggested that the water environment in non-flood season was more suitable for aerobic microorganism growth. Bacterial community was profoundly impacted by DO due to its influence on bacterial activity, and it was a well-recognized phenomenon that DO concentration had a decisive influence in the specific selection of various bacterial lineages. A study by Zhang et al. [45] also investigated that DO and W-TP were significant causes affecting bacterial communities in wetland sediments.

Bai et al. [40] reported that TOC levels had the greatest influence on sediment bacterial communities in Dianchi Lake, China, and Fang et al. [59] and Huang et al. [43] found that T was the dominate factor impacting microbial communities in sediments. The T, DO, and TOC values were highly related with sediment bacteria in the Yellow Sea, China [46]. Furthermore, the environmental elements affecting the bacterial community in Hulun Lake were T, pH, and DO, whereas the environmental factors influencing the fungal community were T, COD, EC, P, and pH [53,60]. It can be seen that microbial communities are strongly associated with their environment. While this study identified a number of environmental factors that influence fungal communities, it was not possible to explain all the observed changes. These unaccounted-for changes could potentially be attributed to environmental factors that have yet to be discovered [53]. There were slight differences in the extent to which environment factors elucidated microbial community variation. For bacterial community, the explanation degree for variation was abundant-community (84.70%) > all-community (45.40%) > rare-community (37.50%). For fungal communities, the explanation degree for variation was abundant-community (48.30%) > rare-community (37.20%) > all-community (35.19%).

### 4.4. Assembly of Sediment Microbial Communities

The spatial and temporal dynamics indicated differences in assembly processes linked to microbial communities. The NCM and null model results further supported this conclusion. The NCM fitted the sediment bacterial community well. The relative contribution made by the stochastic process to the bacterial community of the flood season was lower than the non-flood season; in other words, bacterial community assembly in flood season was more influenced by deterministic factors (such as changes in hydrodynamic conditions, nutrient resource heterogeneity, and interactions between species) [8]. The species dispersal limitation in the sediment bacteria of the non-flood season was higher than the flood season. These results may be due to greater habitat uniformity and river connectivity during flood season than non-flood season [23]. For example, the distance-decay relationship during flood season was weak (Figure 3).

Different from other research results, the assembly process of bacterial community was mainly deterministic, while that of fungal community was mainly stochastic. According to Li et al. [36], bacterial and fungal community assembly in Oxbow lakes were primarily shaped by stochastic processes, which could be caused by the fact that most of the lakes were fully or partially enclosed and therefore the microorganisms were seldom disturbed by outside events. Stochastic processes also dominated bacterial community assembly in an urban river in Ningbo, China [3]. Čačković et al. [61] studied the fungal community of water samples from lower Plitvice Lake, Croatia, and found that community assembly was strongly governed by stochastic processes. Shang et al. [53] investigated fungal communities in the Hulun Lake and found that stochastic processes played an important role compared to deterministic processes. However, a study by Jiao et al. [20] on planktonic and benthic bacteria in a shallow freshwater lake in Nanjing, China, found that deterministic processes dominated bacterial community assembly.

Species interaction, a deterministic process, was a vital factor influencing community aggregation [62]. In this study, findings showed that the microbial networks exhibited substantial seasonal variations, corroborated by the topological characteristics at the node and network levels (Figure 4 and Table 1). The network complexity of the bacterial community was highest in the non-flood season. Research has demonstrated that nutrients are crucial determinants of microbial community network structure [36]. Therefore, alterations to environmental conditions can promote dynamic changes in the microbial network structure. Previous study has shown that stochastic processes contribute to increased species diversity within the environment, promoting interactions among species and enabling the formation of complex microbial networks [63]. However, the relative contribution of stochastic processes from non-flood season to flood season decreased in this study. The decrease in the complexity of flood-season bacterial networks could represent a decrease in the prominence of stochastic processes [59]. The network complexity of the fungal communities was greater during flood season. The increased complexity of fungal networks in flood season may be due to an increase in the importance of deterministic processes, which meant that deterministic processes exerted a stronger impact on fungal communities during flood season.

## 5. Conclusions

The seasonal dynamics of sedimentary microbial communities in the Dali River Basin were examined by amplification sequencing and multivariate statistical analysis. The research results confirmed the correctness of the hypothesis. The diversity (α and β diversity), composition, biomarkers, network structure, and function of microbial community showed temporal dynamic variation, indicating that the microbial community of the Dali River sediment showed seasonal dynamics. The total, abundant, and rare microbial communities had similar geographical patterns and temporal changes. Microbial communities in sediments worked closely together to resist changes in the external environment. Environmental factors caused diversity impacts on microbial communities, and the explanation of bacterial community variation was higher than that of the fungal community, and the explanation of rich microbial community variation was the highest. The deterministic processes governed bacterial community assembly, while stochastic processes governed fungal community assembly. The niche values of bacteria and fungi during non-flood season were higher than flood season. In future studies, the diversity and assembly processes of microbial communities in river sediments in the middle reaches of the Yellow River should be explored on a broader spatiotemporal scale.

## Figures and Tables

**Table 1:** Major topological characteristics of the network structure of community in different seasons.

		Nodes	Links	Modularity	Clustering Coefficient	Average Path Length	Network Diameter	Average Degree	Graph Density	Positive Correlation
Bacteria	Non-flood	132	904	0.377	0.584	3.534	10	13.697	0.105	74.12%
	Flood	43	221	0.33	0.712	2.135	5	10.524	0.257	61.09%
Fungi	Non-flood	109	245	0.678	0.475	5.146	16	4.495	0.042	99.59%
	Flood	149	393	0.833	0.855	1.786	6	5.275	0.036	99.75%

## Data Availability

Data will be made available on request.

## Supplementary Materials

The following supporting information can be downloaded at: https://www.mdpi.com/article/10.3390/microorganisms13020319/s1, S1: Details of collection and determination of water and sediment samples; Figure S1: Variation of physical and chemical properties of environmental factors during non-flood and flood seasons; Figure S2: Variation of composition of bacterial community function during non-flood (a) and flood (b) seasons; Figure S3: Variation of composition of fungal community function during non-flood (a) and flood (b) seasons; Figure S4: Difference of function of bacterial community during non-flood and flood seasons. Bacterial community function with significant differences were identified by STAMP; Figure S5: Environmental drivers of the microbial community function by Mantel test; Table S1: Sampling point coordinate information; Table S2: Difference of function of fungal community during non-flood and flood seasons.
