# Supplementary material for: Mechanisms Driving Seasonal Succession and Community Assembly in Sediment Microbial Communities Across the Dali River Basin, the Loess Plateau, China
Source: Microorganisms. 2025 Feb 1;13(2):319. doi: 10.3390/microorganisms13020319 (PMC11857984; doi:10.3390/microorganisms13020319)
Supplement: Supplementary file 1 [file microorganisms-13-00319-s001.zip › microorganisms-3439984-supplementary.pdf]

Supplementary File Captions

S1. Details of collection and determination of water and sediment samples.

Figure S1. Variation of physical and chemical properties of environmental factors during non-flood and flood seasons.

Figure S2. Variation of composition of bacterial community function during non-flood (a) and flood (b) seasons.

Figure S3. Variation of composition of fungal community function during non-flood (a) and flood (b) seasons.

Figure S4. Difference of function of bacterial community during non-flood and flood seasons. Bacterial community function with significant differences were identified by STAMP.

Figure S5. Environmental drivers of the microbial community function by Mantel test.

Table S1. Sampling point coordinate information.

Table S2. Difference of function of fungal community during non-flood and flood seasons.

S1. Details of collection and determination of water and sediment samples.

Prior to sampling, a HQ30d water quality monitor (HACH, American) was utilized to measure pH, dissolved oxygen (DO), electrical conductivity (EC), and water temperature (T) at the sampling sites. Then, 500 mL sample bottles were rinsed three times with water before collecting the surface water samples for physicochemical properties analysis. Surface sediments at a depth of 0–10 cm was also collected and stored in sterile bags for physicochemical analysis. A total of three replicate samples taken at each sampling site. The three sediment samples were thoroughly mixed and then sealed in 10 mL sterile centrifuge tubes with sealing film for high-throughput sequencing. After collection, all samples were transported back to the laboratory; water samples were stored in a 4°C refrigerator and measured immediately; and the sediment samples in the 10 mL sterile centrifuge tubes were preserved in a –80°C freezer for subsequent DNA extraction. The sediment samples were screened after drying in laboratory to determine their physical and chemical properties.

After drying, the sediment samples were sieved through a 2 mm mesh and their physicochemical properties were analyzed. The organic carbon content in the sediment (S-TOC) was measured using the potassium dichromate titration method and total nitrogen in the sediment (S-TN) was measured using a fully automated Kjeldahl nitrogen analyzer (Foss 8400, Denmark). Total phosphorus (W-TP), total nitrogen (W-TN), and ammonia nitrogen (W-AN) contents in the water samples were measured using an automated discrete analyzer (ADA, Smart Chem 200, Germany). The total phosphorus (S-TP), ammonia nitrogen (S-AN), and available phosphorus (S-SP) contents in the sediments were also measured by an automated discrete analyzer.

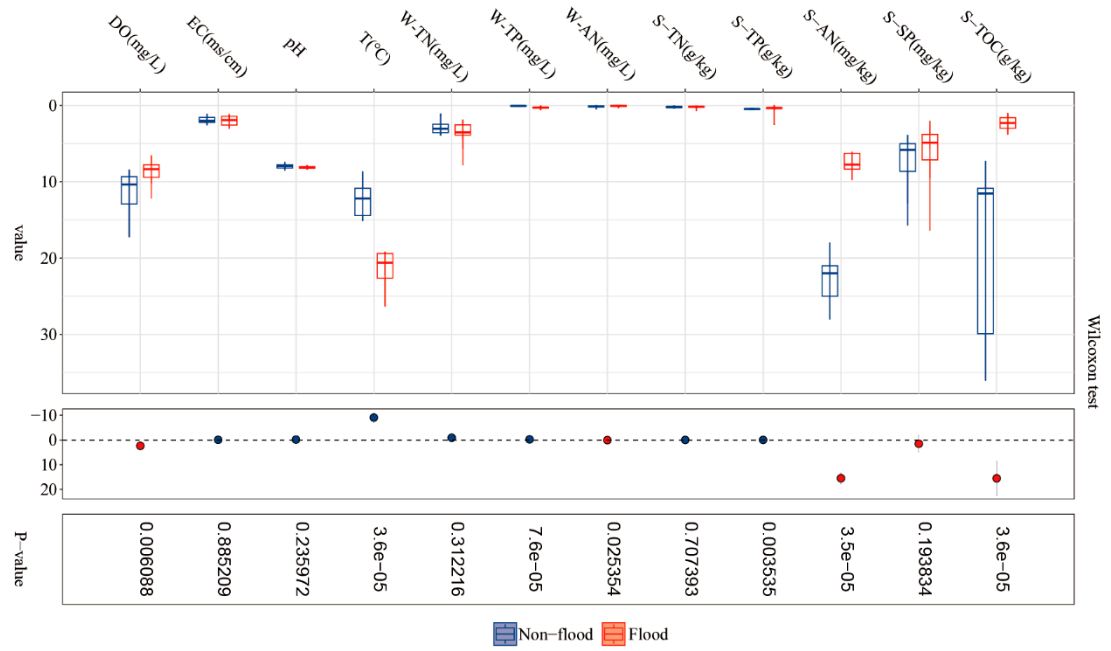

Figure S1. Variation of physical and chemical properties of environmental factors during non-flood and flood seasons.

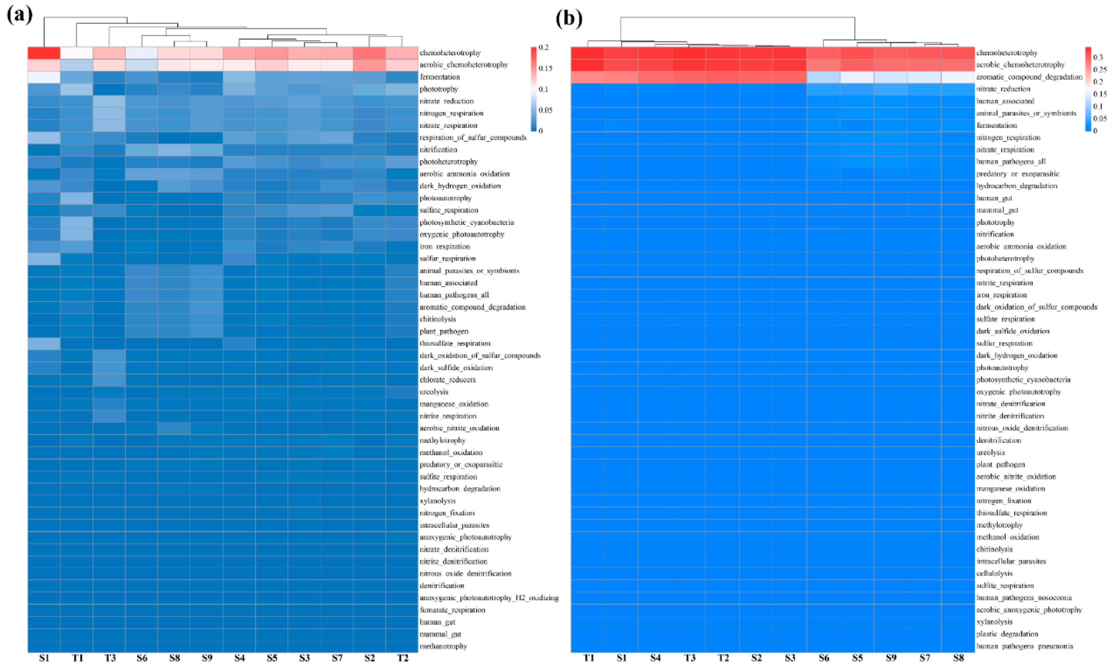

Figure S2. Variation of composition of bacterial community function during non-flood (a) and flood (b) seasons (Top 50).

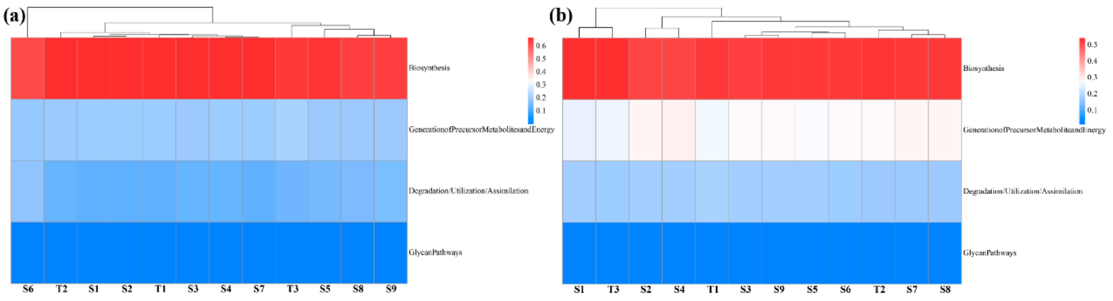

Figure S3. Variation of composition of fungal community function during non-flood (a) and flood (b) seasons.

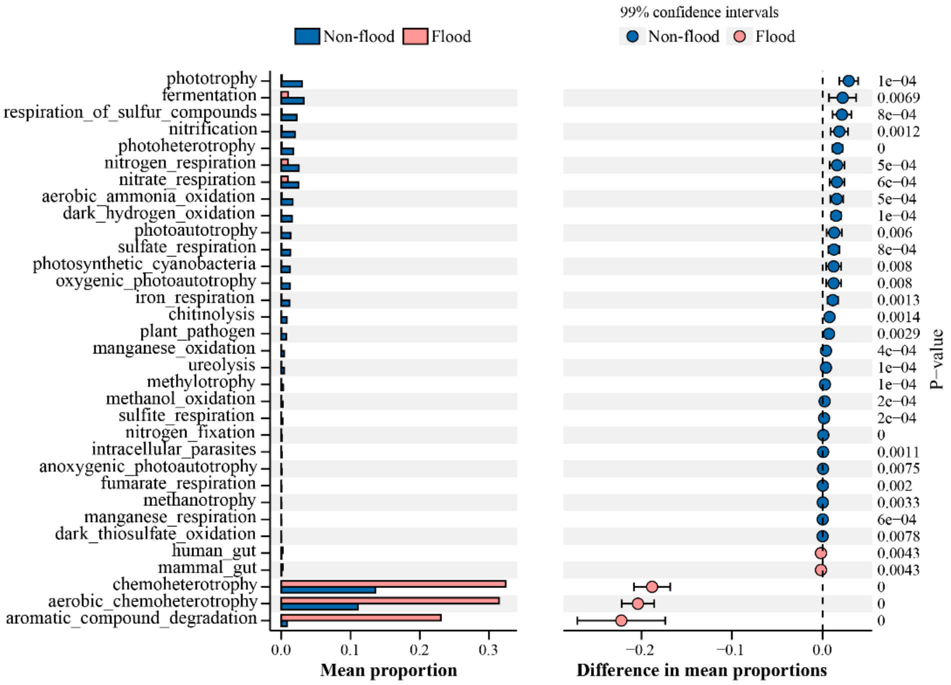

Figure S4. Difference of function of bacterial community during non-flood and flood seasons. Bacterial community function with significant differences were identified by STAMP.

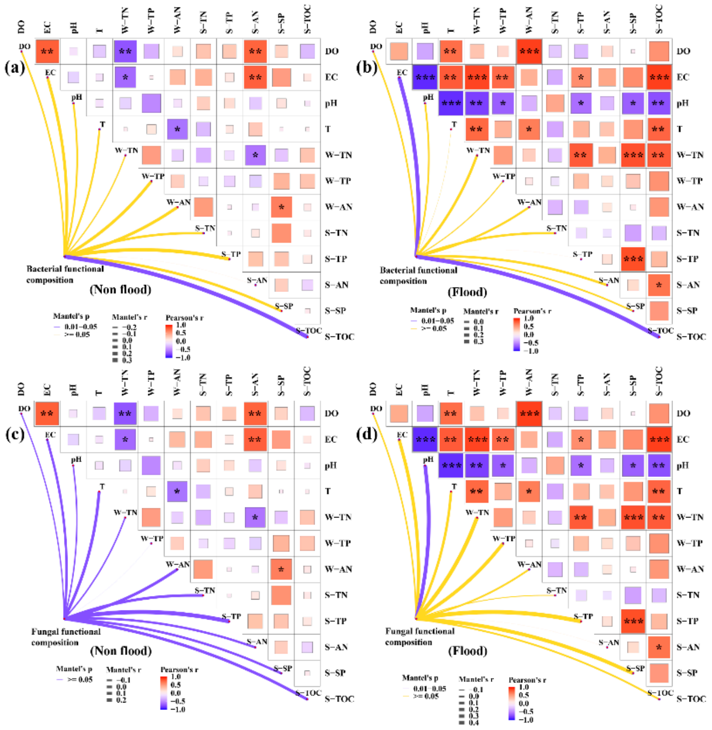

Figure S5. Environmental drivers of the microbial community function by Mantel test.

Table S1. Sampling point coordinate information.

Sample point	Location	Longitude	Latitude
S1	Main stream	110°15′15″	37°30′43″
S2	Main stream	110°6′30″	37°34′40″
S3	Main stream	110°0′18″	37°37′28″
S4	Main stream	109°53′2″	37°35′50″
S5	Main stream	109°46′44″	37°35′13″
S6	Main stream	109°33′21″	37°33′31″
S7	Main stream	109°23′52″	37°28′10″
S8	Main stream	109°14′49″	37°22′4″
S9	Main stream	109°8′6″	37°21′5″
T1	Tributary (Qingyangcha River)	109°11′26″	37°23′8″
T2	Tributary (Chabagou River)	109°55′48″	37°42′3″
T3	Tributary (Xiaoli River)	109°44′41″	37°37′43″

Table S2. Difference of function of fungal community during non-flood and flood seasons.

Level 1	Non-flood	Flood
Biosynthesis	65.14±1.50a	52.65±1.03b
Generation of Precursor Metabolites and Energy	20.54±0.65b	27.92±1.10a
Degradation/Utilization/Assimilation	14.41±1.61b	17.83±0.54a
Glycan Pathways	0.37±0.32b	1.60±0.05a

Note: Different lowercase letters in the same line indicate significant at the 0.05 level.

Table S3. C-score analysis of microbial communities during non-flood and flood seasons.

Project	Bacteria		Fungi	
	Non-flood	Flood	Non-flood	Flood
$C\text{-score}_{obs}$	1.8526	1.1697	1.6615	1.2595
$C\text{-score}_{sim}$	1.8519	1.1662	1.6614	1.2599
P	< 0.001	< 0.001	0.495	0.577
SES	4.9862	5.0643	0.2175	-0.4161
